# Tau and Amyloid-β Peptides in Serum of Patients With Parkinson's Disease: Correlations With CSF Levels and Clinical Parameters

**DOI:** 10.3389/fneur.2022.748599

**Published:** 2022-02-25

**Authors:** Tommaso Schirinzi, Henri Zenuni, Piergiorgio Grillo, Roberta Bovenzi, Gisella Guerrera, Francesca Gargano, Massimo Pieri, Sergio Bernardini, Nicola Biagio Mercuri, Luca Battistini, Giulia Maria Sancesario

**Affiliations:** ^1^Unit of Neurology, Department of Systems Medicine, University of Roma Tor Vergata, Rome, Italy; ^2^European Centre for Brain Research, IRCCS Fondazione Santa Lucia, Rome, Italy; ^3^Department of Experimental Medicine, University of Roma Tor Vergata, Rome, Italy

**Keywords:** Parkinson's disease, blood biomarkers, CSF biomarkers, fluid biomarkers, tau, SiMoA

## Abstract

Relevance of blood-based biomarkers is increasing into the neurodegenerative diseases field, but data on Parkinson's disease (PD) remain still scarce. In this study, we used the SiMoA technique to measure serum content of total tau protein and amyloid-β peptides (Aβ-42, Aβ-40) in 22 PD patients and ten control subjects. Serum levels of each biomarker were correlated with the respective CSF levels in both the groups; in PD patients, also the correlations between serum biomarkers and main clinical parameters were tested (motor, non-motor, cognitive scores and levodopa equivalent daily dose). Serum biomarkers did not exhibit quantitative differences between patients and controls; however, only PD patients had inter-fluids (serum-CSF) associations in tau and amyloid-β-42 levels. Moreover, serum content of tau protein was inversely correlated with cognitive performances (MoCA score). These findings, albeit preliminary, indicate that brain-derived peptides may change in parallel in both peripheral blood and CSF of PD patients, eventually even in association with some clinical features. Further studies are now needed to validate the use of blood-based biomarkers in PD.

## Introduction

One of the most urgent needs for Parkinson's disease (PD) is identification of biomarkers to early diagnose, stratify, and accurately follow-up patients in both interventional and observational frames (1–3).

Cerebrospinal fluid (CSF), because of proximity with the brain, mirrors neuropathological changes, representing an ideal source for reliable disease biomarkers. Indeed, in PD, levels of neurodegeneration-related CSF biomarkers, namely, α-synuclein (α-syn), amyloid-β42 (Aβ42), total tau (t-tau), and phosphorylated-181-tau (p-tau), may have either diagnostic or prognostic value (2–5). However, the invasiveness and difficulties of lumbar puncture prevent repeated samplings and limit the use of CSF biomarkers on a large scale (6, 7).

Recent studies, especially in dementia field, are moving the attention on venous blood as a valuable and easily accessible source of biomarkers, rather satisfactory in terms of diagnostic accuracy (8). Data regarding PD are, instead, still scarce, and clinical values of brain-derived peptides, commonly measured in CSF, have not been tested and validated yet in serums of patients with PD.

Here, we, thus, provided a small pilot, case-control study, measuring the content of amyloid peptides (Aβ42, Aβ40) and total tau in serums of patients with PD and exploring their correlations with corresponding CSF levels and main clinical parameters in order to progress in the use of blood-based biomarkers in PD.

## Methods

### Subjects

The study involved a total of 22 patients with PD and 10 control subjects afferent to the Neurology Unit of Tor Vergata University Hospital (Rome, Italy). PD was diagnosed following the 2015 MDS criteria; controls were subjects without neurodegenerative and/or movement disorders receiving lumbar puncture (LP) for diagnostic purposes (e.g., headache, functional diseases). Subjects with acute and chronic internal/inflammatory/infectious diseases were excluded to prevent biases (3, 9, 10).

For each participant, demographics and medical history were collected. Patients with PD were assessed in the same session of fluids withdrawal, under the effects of antiparkinsonian medication, by means UPDRS III, Non Motor Symptoms Scale (NMSS), Montreal Cognitive Assessment (MoCA), levodopa equivalent daily dose (LEDD) calculation.

The study was in agreement with the ethical principles of Helsinki declaration, and received the approval of the local committee (0026092/2017). Informed written consent was signed by all participants.

### Biomarker Assay

Venous blood sampling and LP were performed following standard procedures, as previously described (10, 11).

Serum biomarkers were measured in all the patients with PD (*n* = 22) and controls (*n* = 10); paired CSF biomarkers were available for *n* = 11 patients with PD and *n* = 7 controls.

Levels of biomarkers in serum were assessed by single-molecule array (SiMoA), an ultrasensitive technology for detection of proteins in blood at sub-femtomolar concentrations (SiMoA HD-1 analyzer; Quanterix Corp., Billerica, MA, United States). All samples were analyzed in duplicate using a SiMoA Human Neurology 3-Plex A assay (N3PA) for simultaneous detection of Aβ42, amyloid-β-40 (Aβ40), and tau following standard procedures. Serum Aβ42/Aβ40 ratio was then calculated.

In CSF, Aβ42, Aβ40, t-tau, and p-tau, were measured by using electrochemiluminescence immunoassay (ECLIA) (LUMIPULSE G β-Amyloid 1-42, LUMIPULSE G β-Amyloid 1-40, LUMIPULSE Total Tau and LUMIPULSE G pTau181 (FUJIREBIO)) (12, 13). CSF Aβ42/ Aβ40 ratio was then calculated. CSF/serum albumin ratio (AR) was evaluated as in routine practice (9).

### Statistical Analysis

Variable distribution was preliminarily examined by Shapiro-Wilk test. Non-normally distributed variables were log10-transformed to allow for statistical calculations.

Data were compared between the groups by parametric (one-way ANOVA) or non-parametric (Mann-Whitney-U test) tests, as appropriate.

In each group, correlations between serum and CSF levels of single biomarkers were tested by simple linear regression. Also, correlations with clinical parameters were explored in the PD group.

A *p* ≤ 0.05 was considered significant. Analysis was conducted blindly with IBM-SPSS-22.

## Results

Table 1 summarizes clinical and biochemical data of the study population. No differences resulted between PD and controls in both serum and CSF biomarkers. In addition, age and gender did not differ.

**Table 1:** Clinical and biochemical data of the study population.

**Variable**	**PD** ***n*** **= 22 (f:54.5%; m:45.5%)**		**Controls** ***n*** **= 10 (f:40%; m:45.5%)**		**Significance**
	**Mean**	**St.dev**.	**Mean**	**St.dev**.	
Age (y)	57.4	11.2	63.3	10.2	ns
Serum t-tau	0.6	0.3	0.6	0.2	ns
Aβ42	2.3	3.2	2.5	3.6	ns
Aβ40	90.1	70.9	89.9	74.1	ns
Aβ42/Aβ40	0.03	0.04	0.02	0.02	ns
CSF t-tau	240.8	137.3	214.1	115.5	ns
p-tau	35.6	18.0	33.7	13.1	ns
Aβ42	669.1	291.2	723.9	277.6	ns
Aβ40	9,863.9	3,160.9	8,276.6	3,728.9	ns
Aβ42/Aβ40	0.07	0.02	0.09	0.04	ns
AR	8.7	3.8	15.3	17.4	ns
Onset (y)	53.8	12.7	na	-	
Duration (y)	5.0	4.8	na	-	
MOCA	23.1	5.2	na	-	
UPDRS III	22.4	14.5	na	-	
NMSS	24.4	21.6	na	-	
LEDD	319.1	418.2	na	-	

*Biomarker concentration in serum and cerebrospinal fluid (CSF) is reported in pg/ml (m, male; f, female; y, years; ns, not significant; na, not applicable; other abbreviations are spelled out in the text)*.

**Figure 1:**
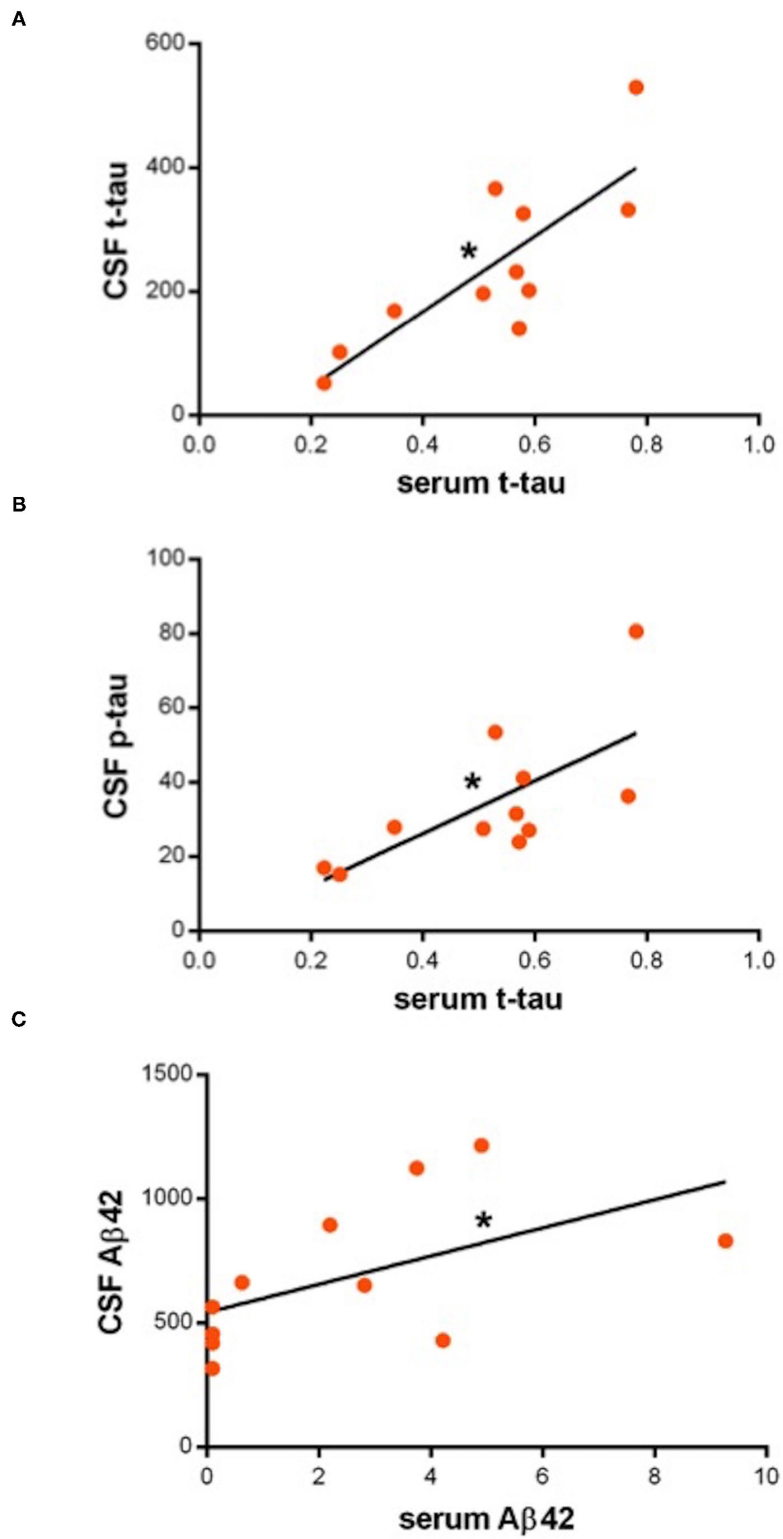
**(A–C)** Dot-plots showing direct correlations between serum and cerebrospinal fluid (CSF) biomarkers. Asterisks indicate statistical significance. Biomarker concentration is expressed in pg/ml.

In the PD group, serum t-tau is directly correlated with both CSF t-tau [F(1, 9) = 16.9, *p* = 0.003, R^2^ = 0.7] (B = 0.001, *p* = 0.003) and p-tau [F(1, 9) = 8.2, *p* < 0.01, R^2^ = 0.47] (B = 0.007, *p* < 0.01); serum Aβ42 is directly correlated with CSF Aβ42 [F(1, 9) = 3.9, *p* = 0.05, R^2^ =0.3] (B = 0.005, *p* = 0.05) (Figure 1); serum Aβ40 is not correlated with CSF Aβ40; serum Aβ42/Aβ40 ratio is not correlated with CSF Aβ42/Aβ40 ratio. None of the serum biomarkers are correlated with AR. Serum tau is inversely correlated with MoCA score [F(1, 10) = 6.3, *p* < 0.03, R^2^ = 0.4] (B = −0.033, *p* < 0.05). No further correlations resulted between the serum biomarkers and the clinical parameters.

In the control group, no correlations emerged between the serum biomarkers and respective CSF levels, AR, or demographic parameters.

## Discussion

This pilot study aimed to assess the use of blood-based neurodegeneration-related biomarkers in PD field, comparing the serum content of t-tau and β-amyloid peptides in patients and controls, and testing correlations with respective CSF levels and main clinical parameters.

As first step, we compared the serum content of t-tau and β-amyloid peptides in patients and controls by means of the SiMoA technique, a methodology already available and validated for detection of sub-femtomolar concentrations of analytes in blood (14). Serum biomarkers did not differ between the two groups. This result could be referred to features of the enrolled population, which may have included patients with mild clinical impairment and minor burden of both amyloidopathy and tauopathy, as the absence of quantitative differences even in CSF biomarkers suggests. However, also, the very small sample size might have had a role.

Then, we performed a correlation analysis between serum and CSF levels of each biomarker on both groups separately. In the controls, levels in the two pools were independent; in the patients with PD, instead, we observed that biomarker concentrations in the two fluids had an association. Specifically, we found correlations for serum t-tau (with both CSF t-tau and p-tau) and serum Aβ42 (with CSF Aβ42), but not for serum Aβ40. Overall, no associations emerged between serum biomarkers and AR (a marker of blood-brain barrier, BBB, integrity) neither in PD nor controls. Collectively, these findings may indicate that the presence of serum biomarkers in patients with PD is not due to disruption of BBB but rather to other specific mechanisms. In fact, dynamics related to brain-blood exchange, the glymphatic system, or exosomes release (15, 16), mediate the passage in bloodstream of molecules involved in central pathology of PD. Indeed, also in Alzheimer's disease (AD), blood contents of tau and Aβ42 basically mirror the respective CSF amounts (17, 18). Conversely, CSF and the serum content of Aβ40 are basically unrelated, suggesting that circulating levels of this peptide, which rises in the brain from metabolic pathways different from those determining senile plaque accumulation, are independent from cerebral amyloidopathy and neurodegeneration, and, instead, modulated by other factors (liver catabolism, peripheral tissues production) (18, 19). Also, systemic conditions, such as coronary artery disease and diabetes, may contribute to serum Aβ40 concentration (20), accounting for detectable levels even in our cohort.

Finally, we sought for correlations between serum biomarkers and PD clinical features, assessed with conventional rating scales. The sole significant association was an inverse one between serum t-tau and MoCA score. In patients with AD, increase in serum tau has been linked to cognitive deterioration and structural changes in many brain areas (17), while clinical correlations of increased blood tau in patients with PD are not established yet. However, higher CSF levels of tau proteins in PD have been measured in patients with cognitive decline (21) or greater brain network disruption (21–23). Accordingly, because of the correlation between serum and CSF t-tau, we could suppose that increase of serum t-tau in patients with PD can reflect widespread degeneration, with subsequent cognitive involvement.

Definitely, there are several study limitations, such as the sample size and cross-sectional design, that impose caution in the interpretation of these data. On the other hand, the homogeneity of the enrolled population may have prevented possible biases due to concurrent conditions (e.g., diabetes, kidney or liver insufficiency), which have to be considered when dealing with blood biomarkers (3). These findings, albeit preliminary, show that brain-derived peptides may change in parallel in both peripheral blood and CSF of PD patients. Molecular events related to neurodegeneration can be thus tracked into a tissue more easily accessible than CSF, which offers similarly potential clinically-relevant information. Further studies on larger cohorts are needed to extend these results and dissect the contribution of peripheral tissues on circulating levels of neurodegeneration-related markers. In addition, the possibility to repeat fluid withdrawals non-invasively may allow the prospective observation of changes in biomarkers levels along PD progression, which is helpful in chronic diseases field more than single time point assessment.

## Data Availability Statement

The raw data supporting the conclusions of this article will be made available by the authors upon reasonable request.

## Ethics Statement

The studies involving human participants were reviewed and approved by Policlinico Tor Vergata EC (0026092/2017). The patients/participants provided their written informed consent to participate in this study.

## Author Contributions

TS and GS conceived the study, collected the data, and wrote the initial draft. HZ, PG, RB, GG, FG, and MP collected and analyzed the data. SB, NB, and LB contributed to data interpretation and revised the manuscript. All authors contributed to the article and approved the submitted version.

## Funding

This study was supported by Italian Ministry of Health — Ricerca Corrente anno 2022.

## Conflict of Interest

The authors declare that the research was conducted in the absence of any commercial or financial relationships that could be construed as a potential conflict of interest.

## Publisher's Note

All claims expressed in this article are solely those of the authors and do not necessarily represent those of their affiliated organizations, or those of the publisher, the editors and the reviewers. Any product that may be evaluated in this article, or claim that may be made by its manufacturer, is not guaranteed or endorsed by the publisher.
